# Cerebrospinal fluid biomarkers in E200K genetic Creutzfeldt–Jakob disease: a national study from an endemic Slovak cohort

**DOI:** 10.3389/fnins.2026.1906444

**Published:** 2026-08-05

**Authors:** Dana Zakova, Silvia Koscova, Katarina Melicherova, Alzbeta Janakova, Zuzana Szarvasova, Dana Kosorinova, Martin Stelzer

**Affiliations:** National Reference Centre for Prion Diseases, Slovak Medical University, Bratislava, Slovakia

**Keywords:** cerebrospinal fluid biomarkers, E200K, genetic CJD, prion disease, RT-QuIC

## Abstract

**Background:**

Genetic Creutzfeldt–Jakob disease (gCJD) is a rare inherited prion disorder caused by pathogenic variants in the *PRNP* gene. Slovakia represents one of the major endemic regions worldwide, with most genetic cases linked to the E200K variant. Reliable cerebrospinal fluid (CSF) biomarkers remain important for diagnosis and surveillance in this setting.

**Methods:**

We analyzed CSF samples from 77 consecutive symptomatic patients with genetically confirmed E200K gCJD referred to the National Reference Centre for Prion Diseases in Slovakia between 2020 and 2024. Total tau (T-tau) concentrations were measured by ELISA and 14-3-3 protein was detected by Western blot as part of the routine diagnostic evaluation throughout the study period. Real-time quaking-induced conversion (RT-QuIC) was introduced in 2023 and was performed prospectively thereafter, while archived frozen CSF samples collected earlier were analyzed retrospectively. Diagnostic sensitivity was evaluated overall and according to the *PRNP* codon 129 genotype.

**Results:**

RT-QuIC showed the highest sensitivity (75/77; 97.4%), followed by T-tau (64/77; 83.1%) and 14-3-3 protein (42/77; 54.5%). RT-QuIC was positive in all methionine/valine heterozygotes (30/30; 100%) and in 45 of 47 methionine homozygotes (95.7%) (*p* = 0.518). In contrast, T-tau and 14-3-3 protein demonstrated significantly higher sensitivity in methionine homozygotes than in heterozygotes (both *p* < 0.0001).

**Conclusion:**

RT-QuIC showed the highest diagnostic sensitivity among the evaluated CSF biomarkers in this endemic cohort. Conventional biomarkers showed marked dependence on *PRNP* codon 129 genotype and may provide complementary diagnostic value.

## Introduction

Prion diseases are fatal neurodegenerative disorders caused by conformational conversion of the normal cellular prion protein (PrPᶜ) into a pathogenic self-propagating isoform (PrPˢᶜ). Genetic Creutzfeldt–Jakob disease (gCJD), caused by pathogenic variants in the *PRNP* gene, represents a minority of human prion disease cases worldwide but demonstrates marked geographic clustering.

Slovakia is considered one of the major endemic regions worldwide for E200K-associated gCJD, with most genetically confirmed cases linked to the *PRNP* p.Glu200Lys variant (Mitrova et al., 1991). This founder effect makes the Slovak population relevant for studies of inherited prion disease biology and diagnostics.

Cerebrospinal fluid biomarkers such as 14-3-3 protein and total tau (T-tau) are widely used supportive markers reflecting rapid neuronal injury, whereas RT-QuIC directly detects prion seeding activity and has substantially improved diagnostic accuracy in prion diseases (Sanchez-Juan et al., 2006; Zerr et al., 2009; Atarashi et al., 2011; Schmitz et al., 2022). Biomarker performance may vary according to host genotype, including the methionine/valine polymorphism at codon 129 of *PRNP*.

We therefore evaluated CSF biomarker performance in a contemporary Slovak E200K gCJD cohort analyzed within a single national reference laboratory framework.

## Materials and methods

### Study design and cohort

We evaluated consecutive CSF samples referred to the National Reference Centre for Prion Diseases, Slovak Medical University, between January 2020 and December 2024 from symptomatic patients with genetically confirmed E200K gCJD.

The cohort included 77 unique patients (32 men, 45 women), with a mean age of 62.7 years (range 39–82 years). All patients carried the pathogenic *PRNP* p. Glu200Lys (E200K) variant. Codon 129 genotype was methionine homozygous (MM) in 47 patients and methionine/valine heterozygous (MV) in 30 patients.

### CSF biomarker analysis

#### Total tau

CSF total tau protein was quantitatively analyzed using a commercially available enzyme-linked immunosorbent assay (INNOTEST^®^ hTAU Ag, Innogenetics, Ghent, Belgium) according to the manufacturer’s instructions. Concentrations >1,300 pg./mL were considered positive, in accordance with established thresholds suggestive of CJD (Sanchez-Juan et al., 2006; Zerr et al., 2009).

#### 14-3-3 protein

Detection of CSF 14-3-3 protein was performed by Western blot using anti-14-3-3 beta primary antibody (ab15260, Abcam, Cambridge, United Kingdom) and peroxidase-conjugated AffiniPure Goat Anti-Rabbit IgG (H + L) secondary antibody (Jackson ImmunoResearch, West Grove, PA, United States).

T-tau and 14-3-3 protein analyses were performed prospectively as part of the routine diagnostic evaluation throughout the study period (see Table 1).

**Table 1:** Diagnostic sensitivity of CSF biomarkers in Slovak E200K gCJD.

Biomarker	Overall	MM	MV
RT-QuIC	75/77 (97.4%)	45/47 (95.7%)	30/30 (100%)
T-tau	64/77 (83.1%)	46/47 (97.9%)	18/30 (60.0%)
14-3-3 protein	42/77 (54.5%)	35/47 (74.5%)	7/30 (23.3%)

#### RT-QuIC

RT-QuIC was performed using a first-generation assay with recombinant hamster prion protein (rPrP) substrate as previously described by Atarashi et al. (2011). The reaction mixture contained 1 × PBS, 170 mM NaCl, 1 mM EDTA, and 10 μM thioflavin T. Reactions were performed in quadruplicate in a FLUOstar Omega microplate reader (BMG LABTECH, Germany) at 42 °C with alternating cycles of shaking (90 s at 900 rpm) and rest (30 s). Fluorescence measurements were recorded every 15 min for a total of 400 cycles. Samples were classified as positive according to the laboratory’s validated standard operating procedure based on run-specific fluorescence thresholds relative to concurrently tested negative controls and positivity in at least two of four replicate wells.

RT-QuIC was introduced into routine diagnostic practice in 2023. The assay was implemented using a validated first-generation protocol employing a commercially available recombinant prion protein substrate available to our laboratory at the time of implementation. The same protocol was subsequently applied to all prospectively and retrospectively analyzed samples to ensure methodological consistency throughout the study period. Therefore, archived CSF samples collected before implementation and stored at −80 °C were analyzed retrospectively, whereas subsequently referred samples were tested prospectively.

### Statistical analysis

Sensitivity was calculated as the proportion of positive results among genetically confirmed cases. Positivity rates between genotype groups were compared using Fisher’s exact test. Total tau concentrations between MM and MV patients were compared using the Mann–Whitney *U* test. A two-sided *p-*value <0.05 was considered statistically significant. Given the observational design and the rarity of E200K gCJD, analyses were primarily descriptive and exploratory.

## Results

### Overall diagnostic sensitivity

RT-QuIC was positive in 75/77 patients (97.4%). T-tau was positive in 64/77 patients (83.1%). 14-3-3 protein was positive in 42/77 patients (54.5%).

### Influence of codon 129 genotype

RT-QuIC was positive in all MV heterozygotes (30/30; 100%) and in 45/47 MM homozygotes (95.7%) (*p* = 0.518).

**Figure 1:**
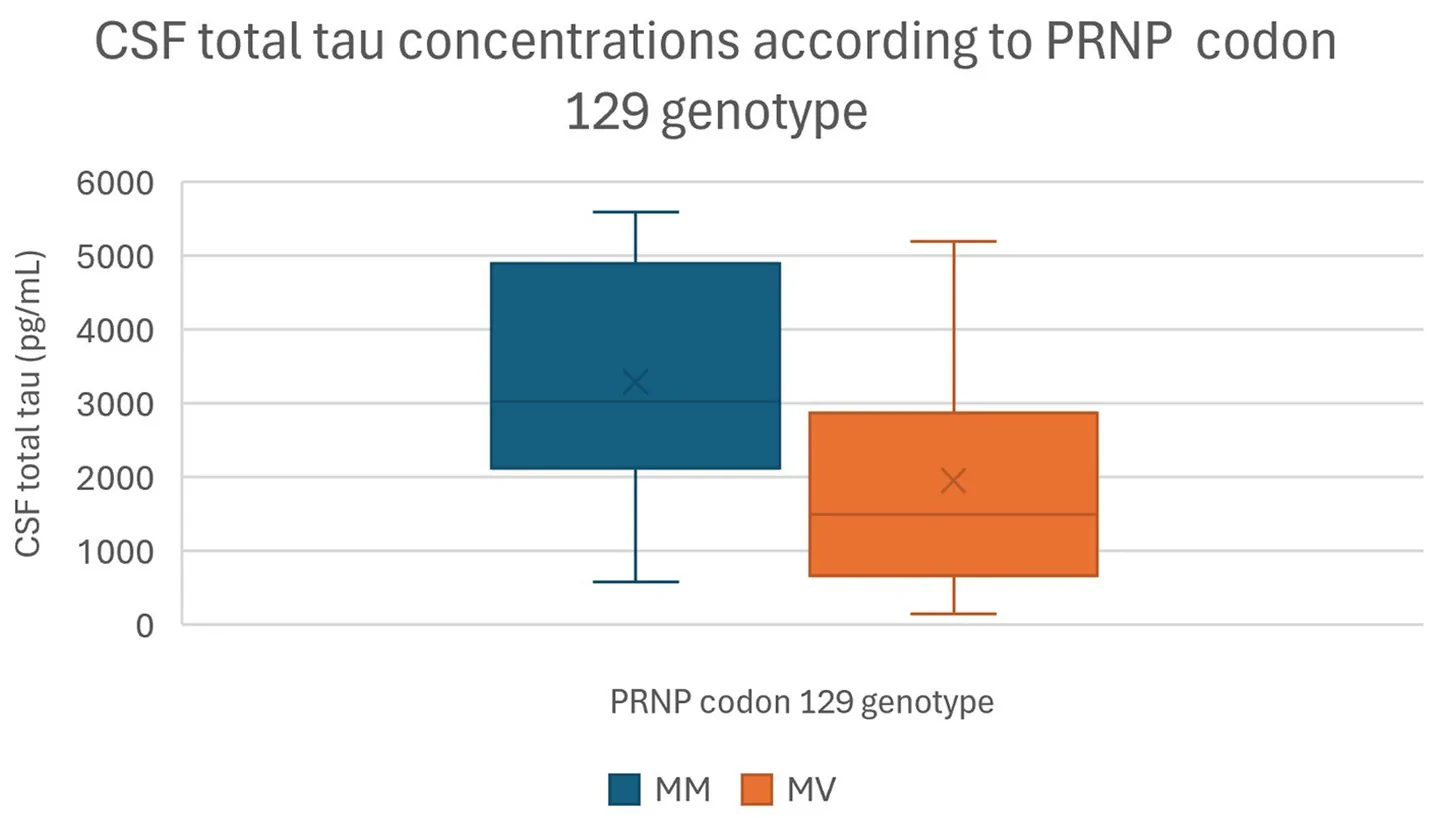
Distribution of CSF total tau concentrations according to PRNP codon 129 genotype in patients with E200K genetic Creutzfeldt–Jakob disease. Total tau concentrations were significantly higher in MM homozygotes than in MV heterozygotes (Mann–Whitney *U* test, *p* < 0.001). The box represents the interquartile range, the horizontal line indicates the median, and whiskers represent the range of values.

T-tau positivity was higher in MM patients than in MV patients (46/47; 97.9% vs. 18/30; 60.0%; *p* < 0.0001). Consistent with this finding, total tau concentrations were significantly higher in MM patients than in MV patients (Mann–Whitney *U* test, *p* < 0.001) (Figure 1).

14-3-3 positivity was also higher in MM patients than in MV patients (35/47; 74.5% vs. 7/30; 23.3%; *p* < 0.0001).

## Discussion

In this national reference laboratory cohort of Slovak patients with E200K gCJD, RT-QuIC showed the highest diagnostic sensitivity among the tested CSF biomarkers, consistent with previous reports (Schmitz et al., 2022).

Two MM patients had negative RT-QuIC results, indicating that false-negative findings may rarely occur even in genetically confirmed disease. Detailed review of these cases did not identify a common clinical or laboratory pattern associated with the negative RT-QuIC results. This observation supports continued multimodal biomarker interpretation in diagnostically challenging cases.

Although RT-QuIC sensitivity appeared numerically higher in MV heterozygotes than in MM homozygotes, the difference was not statistically significant. The relatively small number of MV patients may have limited the statistical power to detect modest genotype-related differences in RT-QuIC sensitivity.

Conventional neuronal injury biomarkers demonstrated marked genotype dependence. Both T-tau and 14-3-3 protein were substantially more sensitive in MM homozygotes than in MV heterozygotes. As both biomarkers primarily reflect neuronal injury rather than prion-specific pathology, the observed differences may be related to genotype-associated variation in disease phenotype and progression.

A major strength of this study is that all samples were analyzed in a single national reference laboratory using consistent methodologies throughout the study period, thereby reducing inter-laboratory variability.

The main limitation of this study is the absence of a control group of non-prion neurological disorders and healthy individuals. Consequently, biomarker specificity, predictive values, and overall diagnostic accuracy could not be assessed, and the findings should be interpreted primarily as estimates of biomarker sensitivity within a genetically confirmed E200K gCJD cohort. In addition, some RT-QuIC analyses were performed on archived frozen CSF samples after assay implementation, which may have introduced preanalytical variability.

Overall, these findings support RT-QuIC as the primary CSF assay for suspected E200K gCJD, with conventional biomarkers providing complementary value. They are also in line with previous Slovak observations on CSF biomarkers in CJD (Koscova et al., 2017).

## Conclusion

RT-QuIC was the most sensitive CSF biomarker in this Slovak E200K cohort. T-tau and 14-3-3 protein remained useful adjunctive markers, particularly in MM homozygotes. These findings support the use of multimodal biomarker assessment in the diagnostic evaluation of suspected E200K gCJD.

## Data Availability

The datasets generated and/or analyzed during the current study are not publicly available due to ethical and privacy restrictions related to sensitive clinical and genetic patient data. They are available from the corresponding author on reasonable request, subject to applicable ethical and institutional regulations.
